# Effect of a 6-month pedometer-based walking intervention on functional capacity in patients with chronic heart failure with reduced (HFrEF) and with preserved (HFpEF) ejection fraction: study protocol for two multicenter randomized controlled trials

**DOI:** 10.1186/s12967-017-1257-x

**Published:** 2017-07-03

**Authors:** Tomas Vetrovsky, Michal Siranec, Jiri Parenica, Martin Griva, Jiri Stastny, Jan Precek, Radek Pelouch, Vaclav Bunc, Ales Linhart, Jan Belohlavek

**Affiliations:** 1 Faculty of Physical Education and Sport, Charles University, Jose Martiho 31, 162 52 Prague 6, Czech Republic; 2 2nd Department of Medicine-Department of Cardiovascular Medicine, 1st Faculty of Medicine, Charles University in Prague and General University Hospital in Prague, U Nemocnice 2, 128 00 Prague 2, Czech Republic; 3 Cardiology Department of University Hospital Brno, Jihlavska 340/20, 625 00 Brno, Czech Republic; 4 Department of Cardiology, Tomas Bata Regional Hospital, Zlin, Havlickovo nabrezi 600, 762 75 Zlin, Czech Republic; 5 Department of Internal Medicine I-Cardiology, University Hospital Olomouc, I. P. Pavlova 185/6, 779 00 Olomouc, Czech Republic; 6 1st Department of Internal Medicine - Cardioangiology, Charles University in Prague - Faculty of Medicine in Hradec Kralove, University Hospital Hradec Kralove, Sokolska 581, 500 05 Hradec Kralove, Czech Republic

**Keywords:** Chronic heart failure, Physical activity, Walking, Functional capacity, Pedometer, 6-min walk test, NT-proBNP

## Abstract

**Background:**

Regular physical activity is recommended for patients with chronic heart failure to improve their functional capacity, and walking is a popular, effective, and safe form of physical activity. Pedometers have shown potential to increase the amount of walking across a range of chronic diseases, but it is unknown whether a pedometer-based intervention improves functional capacity and neurohumoral modulation in heart failure patients.

**Methods:**

Two multicenter randomized controlled trials will be conducted in parallel: one in patients with chronic heart failure with reduced ejection fraction (HFrEF), the other in patients with chronic heart failure with preserved ejection fraction (HFpEF). Each trial will consist of a 6-month intervention with an assessment at baseline, at 3 months, at the end of the intervention, and 6 months after completing the intervention. Each trial will aim to include a total of 200 physically inactive participants with chronic heart failure who will be randomly assigned to intervention or control arms. The 6-month intervention will consist of an individualized pedometer-based walking program with weekly step goals, behavioral face-to-face sessions with a physician, and regular telephone calls with a research nurse. The intervention will be based on effective behavioral principles (goal setting, self-monitoring, personalized feedback). The primary outcome is the change in 6-min walk distance at the end of the 6-month intervention. Secondary outcomes include changes in serum biomarkers levels, pulmonary congestion assessed by ultrasound, average daily step count measured by accelerometry, anthropometric measures, symptoms of depression, health-related quality of life, self-efficacy, and MAGGIC risk score.

**Discussion:**

To our knowledge, these are the first studies to evaluate a pedometer-based walking intervention in patients with chronic heart failure with either reduced or preserved ejection fraction. The studies will contribute to a better understanding of physical activity promotion in heart failure patients to inform future physical activity recommendations and heart failure guidelines.

*Trial registration* The trials are registered in ClinicalTrials.gov, identifiers: NCT03041610, registered 29 January 2017 (HFrEF), NCT03041376, registered 1 February 2017 (HFpEF)

## Background

Chronic heart failure (CHF) is an increasingly costly burden on the health care systems of the developed countries, with a 2.2% prevalence in the American population in 2012 [1]. Impaired functional capacity in CHF patients has detrimental effects on their activities of daily living, health-related quality of life, and ultimately their hospital admission rate and mortality [2–4]. Regular aerobic exercise or physical activity is encouraged in patients with CHF, serving as a safe and effective method of improving their functional capacity and reducing their symptoms [5, 6].

Unfortunately, exercise recommendations are poorly implemented [7] and even those patients who are enrolled in a supervised exercise training program show low adherence [8]. In fact, intense, highly supervised, and structured interventions, such as the program used in the HF-ACTION trial, are not applicable to the wider population of patients with CHF in real-life [9].

As patients are not likely to adhere to such intense exercise programs on a daily basis without supervision, a lifestyle approach can be adopted to promote physical activity. This approach involves the promotion of common daily activities, such as climbing stairs (rather than taking the lift), doing more house work and gardening, engaging in active recreational pursuits, and brisk walking [10].

Walking is a crucial component of the lifestyle approach and it has been described as near perfect exercise [11]. Even walking at a moderate pace of 5 km/h expends sufficient energy to meet the definition of moderate intensity physical activity [12]. Compared with many sports and other recreational pursuits, walking is a popular, familiar, convenient, and flexible form of exercise that can be incorporated into everyday life and sustained throughout the lifespan [13]. Walking is also deemed to be one of the most effective forms of physical activity, with little risk of injury among low-activity populations; it has been used successfully as an intervention to reduce the burden of a number of chronic diseases including hypertension, cardiovascular risk, obesity, and osteoarthritis [14–17].

Pedometers have been commonly employed to provide feedback to patients and have served as a motivational instrument within intervention programs designed to increase activity and improve the quality of life across a range of clinical conditions [17–19]. Results of meta-analyses showed that interventions that have incorporated pedometers have yielded both a significant increase in participants’ physical activity, and a significant decrease in their body mass index and blood pressure [18, 19].

Evidence has shown that utilizing pedometers helps cardiac patients increase their daily physical activity levels [20–25]. However, the large majority of pedometer-based studies in cardiac patients were short-term studies ranging from 3 to 8 weeks, with the exception being one study that lasted 12 months [23]. In addition, most of the studies included fewer than 65 patients with only one study recruiting 110 patients [21] and another recruiting 215 patients [25]. Lastly, and most importantly, none of the studies focused specifically on patients with CHF.

### Rationale and aims

As a whole, the body of literature does not indicate whether using a pedometer-driven walking program increases physical activity in patients with CHF, and if this increase in physical activity translates into improved functional capacity and CHF prognosis. Thus, the main purpose of our randomized controlled multicenter trials is to determine whether a 6-month pedometer-based intervention combining behavioral face-to-face sessions and regular telephone contact improves functional capacity in patients with CHF compared to usual care. We hypothesize that such an intervention would increase an average distance in 6-min walk test (6MWT) by at least 45 m, which is considered as the minimal clinically important difference in patients with CHF [26].

## Methods/design

### Design and settings

Two multicenter randomized controlled trials will be conducted in parallel: one in patients with chronic heart failure with reduced ejection fraction (HFrEF), the other in patients with chronic heart failure with preserved ejection fraction (HFpEF). Patient allocation will be performed as permuted block randomization with a 1:1 ratio. The trials will be conducted across five cardiovascular centers in academic hospitals throughout the Czech Republic:

- General University Hospital, Prague.
- University Hospital, Brno.
- University Hospital, Olomouc.
- University Hospital, Hradec Kralove.
- Tomas Bata Hospital, Zlin.

The study protocol has been approved by the Ethics Committee of the General University Hospital, Prague (20/16 Grant VES 2017 AZV VFN), and the studies will be conducted according to the principles of the Declaration of Helsinki. Eligible patients will be informed of all relevant aspects of the study before enrollment. Participation in the study will be voluntary and will be confirmed via written informed consent. Participants may refuse to participate and will be able to withdraw their consent at any time without reprisal.

**Fig. 1:**
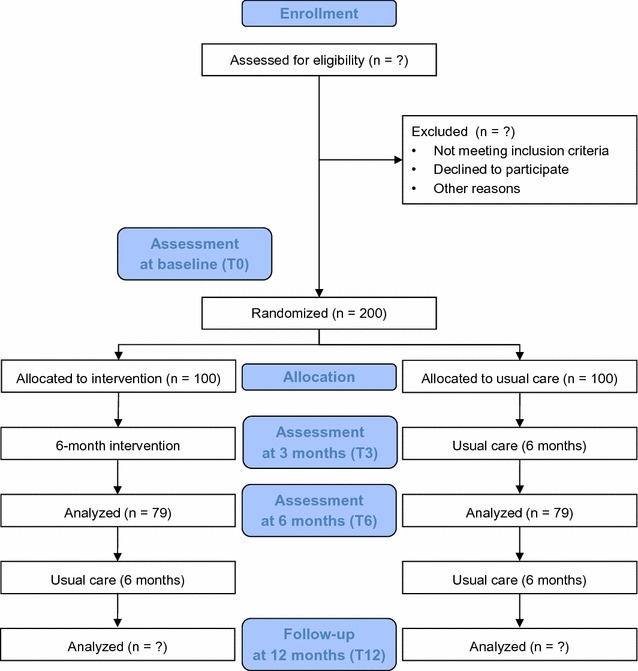
CONSORT 2010 flow diagram

Recruitment has started in April 2017 and the expected completion date for the trials is December 2019. Data will be assessed at baseline (T0), at 3 months (T3), after the 6-month intervention (T6), and at a follow-up visit that will occur 6 months after the cessation of the intervention, which would be 12 months after randomization (T12). A CONSORT flow diagram of the progress through the phases of each study is illustrated in Fig. 1 [27].

This paper is written following the SPIRIT 2013 guidelines [28]. The trials are registered in ClinicalTrials.gov, identifiers: NCT03041610 (HFrEF), NCT03041376 (HFpEF).

## Participants and enrollment

### Eligibility

To participate in one of the trials, patients must comply with all of the following at randomization:

1. Diagnosis of CHF according to the 2016 ESC Guidelines [5] with NYHA class II or III symptoms. Patients will be assigned to one of the two trials:
  A trial that will include patients with heart failure with reduced ejection fraction (HFrEF); i.e. left ventricular ejection fraction (LVEF) <40%;
  A trial that will include patients with heart failure with preserved (HFpEF) or mid-range (HFmrEF) ejection fraction; i.e. fulfilling all of the following criteria: (a) LVEF ≥50 or 40–49%, respectively, (b) the presence of at least one typical symptom and one specific sign of heart failure as defined by the 2016 ESC Guidelines [5], (c) elevated levels of natriuretic peptides (BNP >35 pg/ml and/or NT-proBNP >125 pg/ml), (d) objective evidence of other cardiac functional and structural alterations underlying heart failure.
2. Physically inactive, as determined by the following question: “As a rule, do you do at least half an hour of moderate or vigorous exercise (such as walking or a sport) on five or more days of the week?”. This screening question has a high positive predictive value (86.7%) for identifying individuals who do not achieve the recommended 150 min of moderate level physical activity per week [29].
3. Age ≥ 18 years.
4. Written informed consent obtained before any assessment related to the study.

Patients with both ischemic and non-ischemic etiology of CHF will be included. In patients with ischemic etiology, complete revascularization prior to enrollment into the study will be recommended.

Patients with HFrEF will be required to be on evidence-based standard medication with maximally tolerated dosages. Investigators will be advised to reassess medication dosages before enrolment into the study.

Individuals will be excluded from participation on the following grounds:

1. Signs and symptoms of decompensated heart failure, uncontrolled arrhythmia or effort angina, severe or symptomatic aortic stenosis, or persistent hypotension.
2. Recent (<3 months) myocardial infarction, percutaneous coronary intervention, implantation of an implantable cardioverter defibrillator or bi-ventricular pacemaker, or shocks delivered by the automated implantable cardioverter defibrillator.
3. Co-morbid conditions that would affect adherence to trial procedures (e.g. inflammatory arthritis, active malignancy, renal disease requiring dialysis, uncontrolled diabetes, major depression or other significant psychiatric disorders, cognitive impairment, or significant hearing or visual impairment).
4. Major surgery planned within the next 12 months.
5. Life expectancy shorter than 12 months.
6. Inability to walk from any reason.
7. Baseline 6-min walking distance >450 m. Patients covering more than 450 m in the baseline 6MWT are excluded due to a possible ceiling effect, which has been documented in patients with pulmonary artery hypertension and may also occur in patients with CHF [30, 31].
8. Pregnancy.
9. Failure to perform the 6MWT.

### Sample size

For the purpose of the power analysis, we have chosen a change in six-minute walk distance (6MWD) of 45 m as suggested by a recent review [32]. It has shown that at least moderate effect size of an exercise-based intervention on health-related quality of life appears to be associated with a change in 6MWD greater than 45 m thereby making a change of 45 m the minimum clinically important difference in patients with CHF. It has also suggested that in order to have a reasonable degree of confidence that a change in 6MWD is not due to test–retest variability or measurement error, the amount of change must exceed 43 m [32]. The standard deviation of the response variable in similar populations varies between 38 and 96 m [32]. Therefore, to detect a clinically meaningful change of 45 m on the 6MWT with a power of 80% using a 2-sided 0.05 significance level (alfa) and assuming that the standard deviation is 100 m, 79 subjects in each arm will be needed. To account for an expected attrition rate of 20%, we plan to recruit 100 patients for each arm, resulting in 200 patients for each trial.

### Recruitment and consent

Participants will be identified and recruited during routine clinical visits at each of the participating centers. Potential participants will undergo a screening phase, which will include a review of their medical records for assessment of eligibility. A research team member will evaluate the inclusion and exclusion criteria and will maintain a log where all excluded patients will be recorded, noting the reason why they were excluded.

A research nurse will explain the study in detail to all potentially eligible and interested individuals. Those who will agree to participate following the briefing will be provided with an informed consent form, indicating their full understanding of the study and their protected rights for confidentiality and withdrawal from the study without giving a reason.

### Baseline assessment

After providing consent, patients’ sociodemographic characteristics (ethnicity, marital status, date of birth, education level, and employment status), medical history (heart failure history and etiology, hypertension, hyperlipidemia, etc.), current smoking status and alcohol intake, and current medications will be collected. Then the baseline assessment will be conducted by the research nurse.

During the same visit, participants will be fitted with an ActiGraph accelerometer to measure baseline physical activity. They will be required to wear it for 7 consecutive days and will be instructed to continue their normal physical activities. They will be asked to complete a log of wear time, showing time that the accelerometer is put on and taken off each day and the time and reason that the accelerometer is taken off during the day. The research nurse will then schedule an appointment (at least 8 days after the ActiGraph fitting) to return the device.

### Randomization and blinding

After returning the ActiGraph, individuals will be randomly assigned in a 1:1 ratio to either the control or the intervention group. The randomization will be performed using a central computer-automated randomization system to guarantee adequate allocation concealment. The trials will use a permuted block randomization scheme stratified by center, NYHA class, sex, and age (18–65, ≥66) to ensure equal representation in the groups.

Due to the nature of the study protocols, the process of group allocation cannot be blinded, as the participants and researchers will both be aware of the group allocation due to their active role in the intervention. However, assessments at T3, T6, and T12 will be undertaken by an assessor who is blinded to treatment allocation.

## Intervention and control groups

### Intervention group

The intervention will be delivered over a 6-month period and will consist of: (1) an individualized pedometer-based walking program with weekly step goals, (2) behavioral face-to-face sessions with the physician, and (3) regular telephone calls with the research nurse in between the face-to-face contacts.

The intervention will be based on effective behavioral principles. Goal setting, self-monitoring using the pedometer and an exercise diary, and receiving personalized feedback during the face-to-face sessions and telephone calls are the key behavioral techniques used in the intervention [33].

### Pedometer-based walking program

Following their randomization, participants allocated to the intervention group will receive a triaxial wrist-worn pedometer. The Garmin vívofit (Garmin, Schaffhausen, Switzerland) has been selected as the pedometer of choice to encourage walking behavior of participants in the intervention group as it is currently the most cost effective device that features all of the following characteristics: (1) wrist-worn device as we suppose it might improve patients’ adherence, (2) data can be uploaded online, making data accessible to the research team, (3) a battery life of at least 8 months.

Participants will be asked to wear the pedometer every day, from waking to sleeping, and to upload data online on a weekly basis (eventually with the help of their spouse or younger relatives) at http://www.garminconnect.com. Those unable to upload data will be assisted by the research nurse during face-to-face appointments or during telephone calls. Participants will also be instructed to record the daily number of steps in the exercise diary provided, review the diary at least once a week, and bring both the pedometer and the diary to each appointment.

### Goal setting

Goal setting will be used as an important behavioral component of the intervention. Participants will be instructed not to purposely increase their activity levels during the first week to obtain their habitual daily step count. After the first week, they will be instructed to gradually increase their daily step count during the next 6 weeks to achieve an increase of at least 3000 steps per day above their habitual daily step count at the end of this 6-week period. For the remainder of the intervention period, participants will be encouraged to at least maintain or continue to increase their daily step count.

With an average cadence of 100 steps/min, 3000 steps are equivalent to around 30 min of walking. Participants will be advised to incorporate the walking into their daily routine as either a single 30-min walk or multiple bouts of at least 10 min per bout.

### Face-to-face behavioral sessions

The face-to-face behavioral sessions with a physician will take place during the clinic visits at baseline, at 3 months, and at 6 months. At all sessions, the patients will be reminded of the health benefits of walking, encouraged to integrate walking into their daily routine and implement self-monitoring to achieve their goals. Lastly, they will be reminded to wear the pedometer on a daily basis, regularly upload their data, and maintain their steps-per-day diary. During the session at 3 months, they will be given feedback on their progress based on the diary. During the last face-to-face session at 6 months, participants will return the Garmin vívofit and will be encouraged to maintain or to continue to increase their new level of physical activity without the assistance of the device.

### Telephone calls

The phone calls will be delivered monthly by the research nurse who will have access to the participants’ activity data at http://www.garminconnect.com. The calls will be designed to assess participants’ progress, provide individualized feedback, monitor their adherence, discuss their personal goals and diary, assist them to identify barriers and solutions to physical activity participation, and provide encouragement. The phone calls will be individually tailored based on the current physical activity level and needs of every patient, thus being highly individualized.

### Control group

The participants allocated to the control group will receive their usual care. At the baseline visit, they will neither receive a pedometer nor participate in behavioral session; they will only be educated about the beneficial effects of regular physical activity for patients with CHF and encouraged to increase their physical activity levels. Then, they will be asked to come back for the assessments at 3, 6, and 12 months. During these assessments, no behavioral sessions will take place. The control group participants will not receive any phone contact with the members of the research team.

## Outcome measures

### Assessment schedule

The primary and secondary outcomes detailed below will be assessed at baseline (T0), at 3 months (T3), after the 6-month intervention (T6), and at a follow-up visit 12 months after randomization (T12), as described in Table 1. Regular clinical examinations (NYHA class, vital signs, ejection fraction) will also be performed at T0, T3, T6, and T12. Adverse events will be monitored and recorded throughout the study period.

**Table 1:** Assessment schedule

	T0	T3	T6	T12
Sociodemographic characteristics, medical history	X			
Clinical examination (NYHA class, vital signs)	X	X	X	X
Echocardiography (ejection fraction)	X		X	X
6MWT	X	X	X	X
NT-proBNP	X	X	X	X
hsCRP	X		X	X
Lung ultrasound	X		X	X
Physical activity measured by ActiGraph	X		X	X
Beck depression inventory-II (BDI-II)	X		X	X
36-item short-form health survey (SF-36)	X		X	X
General self-efficacy scale (GSE)	X		X	X
Body weight, height	X	X	X	X
Waist and hip circumference	X		X	X
MAGGIC risk score	X		X	X
Adverse events	X	X	X	X

### Primary outcome

The primary outcome will be the change in distance covered during the 6MWT from T0 to T6. The 6MWT is a practical, simple test that measures the distance that a patient can quickly walk on a flat, hard surface in a period of 6 min. Strong evidence suggests that the 6MWT is responsive to clinical change following cardiac rehabilitation [34]. Lower levels of functional capacity (a distance <300 m during 6MWT) have proven to be predictive of mortality (total or cardiovascular) and morbidity (hospitalization from worsening heart failure) both in patients with asymptomatic left ventricular systolic dysfunction and in those with mild-moderate and advanced heart failure [35].

The test will be performed on a 30-m indoor hallway course with a controlled environment. Patients will be instructed, encouraged, and monitored as recommended in the American Thoracic Society (ATS) guidelines [36]. Briefly, patients will be instructed to walk back and forth in the corridor with the goal to walk as far as possible for 6 min, but they won’t be allowed to run. Only the standardized phrases for encouragement will be used during the test [36].

Although the ATS guidelines suggest that a practice test is not needed in most clinical settings, the guidelines also acknowledge that a learning effect may occur and test performance can be improved during a second trial [36]. In addition, since the ATS guidelines were published, several studies suggest that the test should be duplicated at baseline and at the end of the study [37–39]. Therefore, participants will perform a “practice trial” at all assessment time points; this should refamiliarize the patients with the exercise test and produce valid and reliable results [40]. According to the ATS guidelines, approximately 1 h will separate the practical trial and the measured trial, and the furthest distance will be recorded [36].

### Secondary outcomes

Secondary outcomes include serum biomarker levels, pulmonary congestion assessed by ultrasound, objectively measured physical activity, patient reported outcomes, anthropometric measures, and MAGGIC risk score.

### Biomarker levels

N-terminal pro-B-type natriuretic peptide (NT-proBNP) and high-sensitivity C-reactive protein (hsCRP) have been selected as secondary outcomes as standard, reproducible, and cost-effective assays are available for both biomarkers.

NT-proBNP is the gold standard biomarker for determining the diagnosis and prognosis of CHF. It is used routinely in the clinical management of patients with heart failure as an indicator of heart failure progression, and has a strong prognostic value of death in acute and chronic heart failure [41].

The concentrations of hsCRP are significantly increased with the severity of CHF. An elevated level of hsCRP is an independent predictor of prognosis in CHF, and can provide additional prognostic information for the risk stratification and treatment in patients with CHF [42].

Both markers were used in the HF-ACTION study, and while the exercise training program did not lead to improvements in plasma concentrations of NT-proBNP or hsCRP compared to usual care, serial improvements in NT-proBNP have been associated with increases in peak VO_2_ levels and decreased risk of adverse clinical outcomes [43].

A recent secondary analysis of data from the HF-ACTION trial concluded that exercise therapy was protective for reducing the frequency of membership in the elevated/worsening biomarker pattern of NT-proBNP and hsCRP, indicating that exercise may be helpful in delaying the progression of heart failure [44].

### Lung ultrasound

Lung ultrasound is a novel technique that may allow for the detection and quantification of subclinical pulmonary congestion. B-lines are vertical lines on lung ultrasound which, when quantified, provide a graded measure of pulmonary congestion. A greater number of B-lines have been associated with increased morbidity and mortality [45].

### Physical activity

The ActiGraph GT3X-BT activity monitor (ActiGraph, Pensacola, FL, USA) will be used to objectively measure average daily step count measured over 7-day periods at T0, T6, and T12. The ActiGraph has been found to be reliable and valid in laboratory testing and for the measurement of everyday activities [46].

The activity monitor will be affixed to an elastic belt and worn on the waist for 7 full days during waking hours, except when swimming or bathing. Participants will also be asked to complete an activity monitor log to indicate when the monitor was removed. For the purpose of these studies, valid wear time will be determined as at least 8 h of activity on at least 5 of the 7 days.

### Patient reported outcomes

Patient reported outcomes include symptoms of depression (BDI-II), health-related quality of life (SF-36), and self-efficacy (GSE).

The Beck Depression Inventory-II (BDI-II) is a 21-item, self-reported measure of depressive symptoms using a 0–3 scale [47]. The BDI-II has excellent psychometric properties and has been widely studied in cardiac patients, including the HF-ACTION trial [48].

The SF-36 is a validated measure of health-related quality of life that assesses mental and physical health [49]. It consists of 36 questions divided into eight individually analyzed dimensions: vitality, physical functioning, bodily pain, general health perceptions, physical role functioning, emotional role functioning, social role functioning, and mental health.

Self-efficacy is the degree of confidence an individual has in their ability to perform behavior under specific circumstances [50] and it plays an important role in the adoption and maintenance of physical activity in older adults [51]. Self-efficacy will be assessed by the Czech version [52] of the General Self-Efficacy Scale (GSE) [53].

### Anthropometric measures

Height, body weight, and waist and hip circumference will be measured by an assessor blinded to the participants’ group allocation. Participants will be asked to remove any footwear and to wear only light clothing for anthropometric measurements. Height will be measured using a stadiometer to the nearest 0.1 cm. Body weight will be measured on a calibrated electronic scale to the nearest 0.1 kg. Body mass index will be calculated by dividing the body weight (kg) by the square of the height (m^2^). Waist and hip circumference will be recorded with a measurement tape to the nearest 0.1 cm, according to established protocols [54].

### MAGGIC risk score

The MAGGIC risk score is a simple method to predict survival in heart failure patients. It includes 13 highly significant independent predictors of mortality: age, ejection fraction, NYHA class, serum creatinine, diabetes, beta-blocker usage, systolic blood pressure, body mass, time since diagnosis, current smoking status, presence of chronic obstructive pulmonary disease, gender, and usage of ACE-inhibitors or angiotensin-receptor blockers [55]. The MAGGIC risk score calculator is available at http://www.heartfailurerisk.org.

### Adverse events

Adverse events will be monitored and recorded throughout the study period. Data regarding falls, injuries, musculoskeletal problems, major cardiovascular disease events, and any other events potentially related to implementation of the study protocol will be collected at T3, T6, and T12.

### Data analysis

The primary analysis will compare the change in 6MWD from T0 to T6 between the intervention and control groups. The analysis of the primary and secondary outcomes will be undertaken on an intention-to-treat basis. Primary and secondary measures will be compared between the two groups using two-sample t tests or their non-parametric alternative, if necessary. A *p* value of ≤0.05 will be considered as statistically significant and all tests will be two tailed.

Furthermore, two-sided 95% confidence intervals will be constructed to describe the differences. Differences at T12 will be tested only if the 6-month intervention is shown to be successful at the T6 measurements. Baseline characteristics will be compared between the intervention and control groups. If significant differences will be demonstrated, the measure will be added into statistical models as a covariate. If significant differences will be demonstrated in more measures which are correlated, only one measure will be added as a covariate in order to avoid multicollinearity.

The impact of missing data will be assessed using a sensitivity analysis and missing data will be imputed using a multiple imputation procedure, where necessary. All statistical analyses will be performed using SAS version 9.4 (SAS Institute Inc., Cary, NC, USA) and the statistical package *R*.

## Discussion

Despite the proven benefits of regular physical activity in heart failure patients, their participation rates in cardiac rehabilitation programs remain low. The purpose of these studies is to evaluate the effect of pedometer-based walking intervention combining regular face-to-face appointments and telephone contacts on functional capacity in patients with CHF using a multicenter randomized controlled approach. Such studies have not been performed before.

Other strengths of our studies include: (1) The pragmatic design of the study, when only regular physicians and nurses of the cardiology department without any extensive behavioral training deliver the intervention, makes it (if beneficial) applicable to routine clinical practice. (2) Unlike most pedometer-based interventions so far, the device used in our study synchronizes easily with the server, making the step data in minute epochs for the 6-month period of the intervention available for an auxiliary analysis of physical activity patterns of heart failure patients. (3) While most walking interventions to date employed waist-worn pedometers and accelerometers, we have chosen a new wrist-worn device as this might improve adherence to wearing it. (4) The step goal for each patient is set individually, based on their baseline physical activity levels which again increases patient’s commitment and their willingness to achieve it.

The HF-ACTION trial, the largest randomized trial in CHF patients to date, compared 3-month exercise training program with usual care in 2331 heart failure patients. When analyzed per protocol, exercise training led only to a non-significant 7% reduction in all-cause mortality or hospitalization. Only after adjustment for pre-specified major prognostic factors, the composite primary endpoint was significantly reduced by 11% (*p* = 0.03) [9]. This lower than expected effect can be partially attributed to a low level of adherence to the prescribed training regimen [8]. Thus, a potential challenge of our studies will be to maximize adherence to the proposed intervention. We aim to address the challenge by ensuring frequent contact with the clinical staff and employing effective behavioral strategies that enhance patient self-efficacy, such as realistic goal setting, self-monitoring, feedback, and positive encouragement [56].

Our studies will contribute to a better understanding of physical activity promotion in heart failure patients. If shown to be beneficial, it will indicate that using pedometers provides enough feedback for patients to adhere to a program without the overbearing supervision of a rigid, intense exercise program, encourage clinicians to prescribe exercise and physical activity as an integral part of heart failure management, and improve health outcomes for heart failure patients.

## Abbreviations

CHF: chronic heart failure
HFrEF: heart failure with reduced ejection fraction
HFpEF: heart failure with preserved ejection fraction
HFmrEF: heart failure with mid-range ejection fraction
LVEF: left ventricular ejection fraction
6MWD: six-minute walk distance
6MWT: six-minute walk test
ATS: American Thoracic Society
ESC: European Society of Cardiology
NT-proBNP: N-terminal pro-B-type natriuretic peptide
BNP: B-type natriuretic peptide
hsCRP: high-sensitivity C-reactive protein
GSE: General Self-Efficacy Scale
BDI-II: Beck Depression Inventory-II
SF-36: Medical Outcome Study Short Form-36
NYHA: New York Heart Association
MAGGIC: Meta-Analysis Global Group in Chronic Heart Failure
